# Supplementary material for: The Diagnostic Accuracy of Magnetic Resonance Imaging Versus Transvaginal Ultrasound in Deep Infiltrating Endometriosis and Their Impact on Surgical Decision-Making: A Systematic Review
Source: Diagnostics (Basel). 2025 Nov 12;15(22):2856. doi: 10.3390/diagnostics15222856 (PMC12651815; doi:10.3390/diagnostics15222856)
Supplement: Supplementary file 1 [file diagnostics-15-02856-s001.zip › diagnostics-3967811-supplementary.pdf]

Supplementary Tables:

Table S1. Excluded Studies with Reasons

Study (Author, Year)	Reason for Exclusion
Alborzi et al. (2018)	Did not compare MRI and TVUS in the same cohort; modalities assessed independently
Alborzi et al. (2022)	Focused only on rectal DIE; no whole-pelvis or anterior disease comparison; no surgical impact discussed
Brusic et al. (2019)	MRI only; no TVUS comparator; focused on predicting colorectal surgeon need
Desplats et al. (2019)	Used endoscopic ultrasound; did not include MRI or TVUS comparison
El-Maadawy et al. (2021)	TVUS only; no MRI data; no comparator design
Elsadawy et al. (2023)	MRI only; did not include TVUS comparator; retrospective correlation with ENZIAN and laparoscopy
Enzelberger et al. (2022)	Did not compare diagnostic modalities; focused on preoperative application of the Enzian classification system
Rousset et al. (2021)	MRI only; aimed to predict need for segmental resection, not compare with TVUS
Scardapane et al. (2017)	Used MR colonography; no TVUS comparator; predictive model of bowel resection only

Table S2. Boolean Search Structures

PubMed Search Structure<br><br>(("deep infiltrating endometriosis"[Title/Abstract] OR DIE[Title/Abstract]) AND (("magnetic resonance imaging"[Title/Abstract] OR MRI[Title/Abstract]) AND ("transvaginal ultrasound"[Title/Abstract] OR TVUS[Title/Abstract]))) OR (("deep infiltrating endometriosis"[Title/Abstract] OR DIE[Title/Abstract]) AND ("magnetic resonance imaging"[Title/Abstract] OR MRI[Title/Abstract] OR "transvaginal ultrasound"[Title/Abstract] OR TVUS[Title/Abstract]) AND ("surgical planning"[Title/Abstract] OR "surgical decision-making"[Title/Abstract] OR "operative strategy"[Title/Abstract] OR "preoperative imaging"[Title/Abstract]))) AND ("2015/01/01"[Date - Publication] : "2025/12/31"[Date - Publication]) AND English[Language]
Scopus Search Structure<br><br>((TITLE-ABS ("deep infiltrating endometriosis") OR TITLE-ABS (die)) AND (((TITLE-ABS ("magnetic resonance imaging") OR TITLE-ABS (mri)) AND (TITLE-ABS ("transvaginal ultrasound") OR TITLE-ABS (tvus))) OR ((TITLE-ABS ("magnetic resonance imaging") OR TITLE-ABS (mri) OR TITLE-ABS ("transvaginal ultrasound") OR TITLE-ABS (tvus)) AND (TITLE-ABS ("surgical planning") OR TITLE-ABS ("surgical decision-making") OR TITLE-ABS ("operative strategy") OR TITLE-ABS ("preoperative imaging"))))) AND PUBYEAR > 2014 AND PUBYEAR < 2026
Embase Search Structure<br><br>('deep infiltrating endometriosis'/exp OR 'deep infiltrating endometriosis':ti,ab OR die:ti,ab) AND (('magnetic resonance imaging'/exp OR 'magnetic resonance imaging':ti,ab OR mri:ti,ab) AND ('transvaginal ultrasonography'/exp OR 'transvaginal ultrasound':ti,ab OR tvus:ti,ab) OR (('magnetic resonance imaging'/exp OR 'magnetic resonance imaging':ti,ab OR mri:ti,ab OR 'transvaginal ultrasonography'/exp OR 'transvaginal ultrasound':ti,ab OR tvus:ti,ab) AND ('surgical planning':ti,ab OR 'surgical decision making':ti,ab OR 'operative strategy':ti,ab OR 'preoperative imaging':ti,ab))) AND [english]/lim AND [2015-2025]/py AND [humans]/lim

Table S3. QUADAS-2 Table

Study	Patient Selection	Index Test	Reference Standard	Flow & Timing	Applicability Concerns
Aas-Eng et al. (2023)	Low	Unclear	Low	Low	Low
Bielen et al. (2020)	Low	Low	Low	Low	Low
Guerriero et al. (2017)	Unclear	Unclear	Low	Low	Low
Indrielle-Kelly et al.	Low	Low	Low	Low	Low
Philip et al. (2020)	Low	Unclear	Low	Unclear	Low
Puri et al. (2022)	High	High	Low	Unclear	High
Roditis et al. (2023)	Unclear	Unclear	Low	Low	Low
Sloss et al. (2022)	Unclear	Unclear	Low	Unclear	Low
Zaidi et al. (2023)	Low	High	Low	Unclear	High

Table S4. Newcastle–Ottawa Scale (NOS) quality assessment of included studies

Study	Design / Setting	Selection (0–4)	Comparability (0–2)	Outcome (0–3)	Total (0–9)	Overall quality
Bielen et al., 2020 (Belgium; MRI vs TVUS for DIE compartments)	Prospective / single centre	4 (clear inclusion; same cohort; surgery reference)	2 (MRI vs TVUS within-subject)	2 (outcome clearly defined, but no long follow-up)	8	High
Guerriero et al., 2018 (2D/3D US vs MRI)	Prospective comparative	4 (consecutive women with suspected DIE; surgical gold standard)	2 (direct comparison of modalities)	2 (clear outcome, but no follow-up)	8	High
Indrielle-Kelly et al., 2020 (IDEA-based mapping, US vs MRI)	Prospective / protocolised	4 (well-defined population; reference surgery)	2 (IDEA framework; same patients)	2 (outcomes clearly reported)	8	High
Aas-Eng et al., 2023 (rectosigmoid, TVUS vs MRI, prospective)	Prospective / tertiary centre	4 (all women discussed at MDT, then surgery)	2 (head-to-head TVUS/MRI)	2 (histology as outcome; interval reported)	8	High
Philip et al., 2020 (3D rectosonography vs MRI, 101 pts)	Prospective cohort	4 (consecutive symptomatic women; surgery)	2 (two modalities same cohort)	2 (clear outcome, no long follow-up)	8	High
Puri et al., 2022 (MRI vs TVUS; Indian cohort)	Prospective / single centre	3 (possible non-consecutive recruitment)	2 (same patients had both tests)	2	7	High
Roditis et al., 2023 (clinical exam + TVUS + MRI combination)	Prospective	3 (specialist centre, possible selection)	2 (within-patient comparison)	2	7	High
Sloss et al., 2022 (Australia, rectosigmoid depth)	Retrospective analysis of prospectively collected cohort	3 (convenience/MDM population)	2 (both modalities, same cases)	2 (histology reference)	7	High
Zaidi et al., 2023 (Pakistan, 90 women, TVUS vs MRI vs laparoscopy)	Prospective, LMIC, single centre	3 (consecutive but some exclusions; local sample)	1 (limited adjustment, but same women got both tests)	2 (laparoscopy + histology as reference)	6	Moderate–high
Abbreviations: MRI; Magnetic Resonance Imaging, TVUS; Transvaginal Ultrasound						

Table S5. Summary of Technical Protocols across included studies

Author (Year)	TVUS technique	Bowel prep	Standardisation IDEA/ENZIAN	MRI sequences
**Aas-Eng et al. (2023)**	2D TVUS with LAVD mapping	No	IDEA guidance used for LAVD positioning	3D T2W, high-res 2D T2W, T1 Dixon, fat-suppressed T1; antispasmodic & enema used
**Bielen et al. (2020)**	Expert 2D TVUS + IVU + DCBE	Yes (for DCBE)	IDEA referenced, but not strictly applied	High-res T2W and T1W with fat suppression, vaginal and rectal gel, IV gadolinium
**Guerriero et al. (2017)**	2D and 3D TVUS	Yes (fasting + antispasmodic)	Systematic compartment-based scanning	T1, T2 with and without fat suppression, gadolinium contrast
**Indrielle-Kelly et al. (2020)**	2D TVUS using full IDEA protocol	No	IDEA applied to US, MRI, and surgical mapping	T2, T1 with fat suppression, adapted IDEA protocol
**Philip et al. (2020)**	3D rectosonography with water contrast	Yes (enema and intrarectal water)	Not explicitly stated	T2W and T1W, vaginal and rectal contrast used
**Puri et al. (2022)**	Standard 2D TVUS	Not mentioned	No	T1W, T2W, T1 fat-sat, contrast-enhanced sequences
**Roditis et al. (2023)**	2D expert TVUS	No	dPEI classification used for MRI only	3DT2W, T1 with/without fat suppression, no vaginal/rectal gel
**Sloss et al. (2022)**	Standard 2D TVUS by specialists	Fleet enema	No mention	T2W, no contrast, no vaginal/rectal gel
**Zaidi et al. (2023)**	Standard 2D TVUS	Not mentioned	Not used	T1W, T2W, fat-sat, with contrast where needed

Abbreviations:
MRI; Magnetic Resonance Imaging, TVUS; Transvaginal Ultrasound
LAVD; Lesion to anal verge distance, IVU; Intravenous Urogram
DCBE; Double Contrast Barium Enema, dPEI; deep Pelvic Endometriosis Index

Table S6. Diagnostic performance of TVUS and MRI for DIE with 95 % confidence intervals (CIs) where available.

Study (Year)	Site(s) Assessed	Modality	Sensitivity (95 % CI)	Specificity (95 % CI)	PPV (95 % CI)	NPV (95 % CI)	Notes
Guerriero et al., 2018	Intestinal DIE	2D US	84.8 (76.2–93.5)	87.1 (80.3–93.9)	82.4 (73.3–91.4)	89.0 (82.6–95.4)	Full 95 % CIs reported in study
		3D US	89.4 (82.0–96.8)	93.5 (88.6–98.5)	90.8 (83.7–97.8)	92.6 (87.2–97.9)	
		MRI	92.4 (86.0–98.8)	94.6 (90.0–99.2)	92.4 (86.0–98.2)	94.6 (90.0–99.2)	
Indrielle-Kelly et al., 2020	Multisite DIE (USL, rectosigmoid, bladder etc.)	TVUS	78 (69–86)	97 (94–99)	93 (90–95)	91 (89–94)	95 % CIs reported per site
		MRI	84 (79–91)	96 (91–99)	89 (81–94)	94 (90–98)	
Philip et al., 2020	Rectosigmoid DIE	3D RSG (TVUS-based)	0.93 (0.84–0.98)	0.95 (0.83–0.99)	0.97 (0.87–0.99)	0.91 (0.79–0.96)	Table 2 in article
		MRI	0.87 (0.75–0.94)	0.90 (0.77–0.97)	0.93 (0.84–0.97)	0.82 (0.71–0.90)	
Roditis et al., 2023	Multisite DIE (USL, vagina, rectosigmoid, bladder)	TVUS	61.5–87.5 (range across sites)	83.5–99.4	88.9–100.0	69.4–98.8	95 % CIs reported per site (Table 2)
		MRI	84.6–98.0	83.3–99.4	91.7–100	88.6–98.8	
Zaidi et al., 2023	Multisite DIE	TVUS	0.77 (0.66–0.85)	0.70 (0.48–0.86)	0.90 (0.80–0.95)	0.47 (0.30–0.64)	Wilson 95 % CIs calculated from TP/TN data
		MRI	0.91 (0.83–0.96)	0.85 (0.64–0.95)	0.96 (0.88–0.99)	0.74 (0.54–0.88)	

Abbreviations:
CI; Confidence Intervals, PPV; Positive Predictive Value, NPV; Negative Predictive Value

Figure S1. Radar charts comparing the average diagnostic performance of MRI (orange) and TVUS (blue) for DIE across anatomical compartments

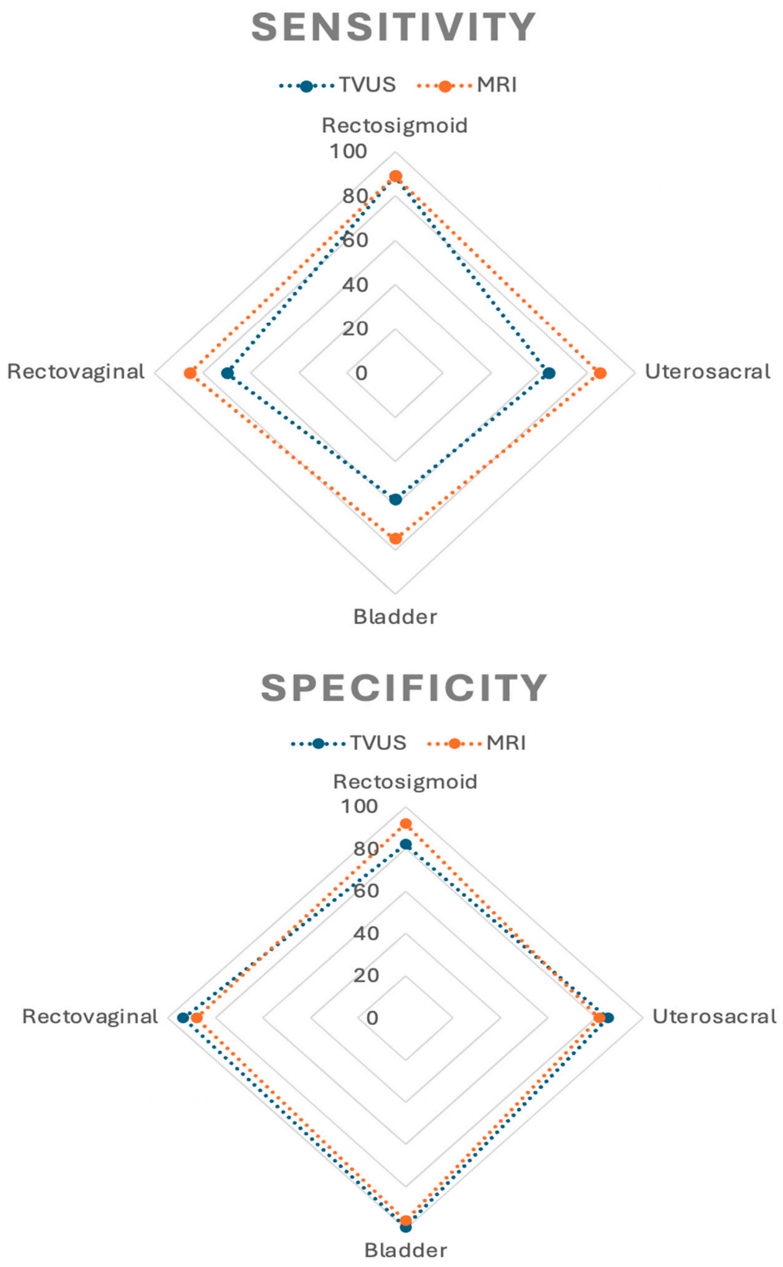

MRI demonstrates consistently higher sensitivity, particularly for uterosacral and bladder disease, while specificity is similar for both modalities.
